# Left-handed sperm removal by male *Calopteryx* damselflies (Odonata)

**DOI:** 10.1186/2193-1801-3-144

**Published:** 2014-03-17

**Authors:** Kaori Tsuchiya, Fumio Hayashi

**Affiliations:** Department of Biology, Tokyo Metropolitan University, Minamiosawa 1-1, Hachioji, Tokyo 192-0397 Japan

**Keywords:** Handedness, Male genitalia, Morphological asymmetry, Sexual selection, Sperm competition

## Abstract

Male genitalia in several insect species are asymmetry in right and left shape. However, the function of such asymmetric male genitalia is still unclear. We found that the male genitalia of the damselfly *Calopteryx cornelia* (Odonata: Calopterygidae) are morphologically symmetric just after emergence but asymmetric after reproductive maturation. Males remove rival sperm stored in the female bursa copulatrix (single spherical sac) and the following spermatheca (Y-shaped tubular sac) prior to their own ejaculation to prevent sperm competition. Males possess the aedeagus with a recurved head to remove bursal sperm and a pair of spiny lateral processes to remove spermathecal sperm. The right lateral process is less developed than the left, and sperm stored in the right spermathecal tube are rarely removed. Experiments involving surgical cutting of each lateral process demonstrated that only the left process functions in spermathecal sperm removal. Thus, males of *C. cornelia* are left-handed in their sperm removal behaviour at copulation.

## Background

Bilaterally symmetric organisms are common, and so asymmetry is a pervading phenomenon. Recent studies have highlighted the adaptive significance of asymmetric organs and their consequent behaviours; for example, laterally biased male-male fighting in fish (Takeuchi et al. 2010), left/right-handed scale-eating in fish (Hori 1993), right-handed snail-eating in snakes (Hoso et al. 2007) and in larval water beetles (Inoda et al. 2003), and left/rightward predator-avoiding jumping in shrimp (Takeuchi et al. 2008), crayfish (Tobo et al. 2012) and cuttlefish (Lucky et al. 2012). Insects are the most divergent animal group and their taxonomic literature has provided details on many cases of asymmetric genitalia (reviewed by Huber et al. 2007;Schilthuizen 2013). Asymmetries comprise two major categories; directional asymmetry in which individuals in a population are structurally or behaviourally asymmetrical toward one side, and antisymmetry in which left- and right-handed individuals are equally frequent in a population (Palmer 1996). Insect genital asymmetries are predominantly directional and limited to the male, and antisymmetric insect genitalia are apparently very rare (Huber et al. 2007;Schilthuizen 2013). The genital asymmetries may be related to one-sided mating position (or abdominal twists), a split of function (grasp, transfer sperm, stimulate etc.) between left and right sides, and internal space constraints that favour asymmetric placement of internal organs (Huber et al. 2007;Schilthuizen 2013). However, their adaptive significance has been examined in only a few studies; right-handed insemination by earwigs (Kamimura 2006) and mating success in *Drosophila pachea* (Lang and Orgogozo 2012).

Sperm competition, when the ejaculates of two or more males compete to fertilise an ovum, could cause genital divergence via male fertilisation success. Significant variance in fertilisation success for males is attributable to differences in male copulation behaviour and male genital morphology (reviewed by Eberhard 1996;Córdoba-Aguilar 2010;Simmons 2014). This adds to the growing number of empirical studies that have provided evidence for sexual selection as a pervasive force, shaping the evolution of genitalia. Sperm displacement by males is one mechanism of avoiding sperm competition in insects (Simmons 2001). Such sperm displacement is well known in Calopterygidae damselflies (Odonata) (Córdoba-Aguilar and Cordero Rivera 2005). Males of these damselflies possess a unique aedeagus with a recurved head and two spiny lateral processes, while females have two sperm-storage organs, a spherical bursa copulatrix and a tubular Y-shaped spermatheca. Previous studies demonstrated that the recurved head removes bursal sperm, whereas the lateral processes potentially remove spermathecal sperm (Waage 1979;Córdoba-Aguilar et al. 2003;Hayashi and Tsuchiya 2005;Tsuchiya and Hayashi 2008).

We found that the male genitalia of this group of damselflies *Calopteryx cornelia* (Odonata: Calopterygidae) are morphologically asymmetrical and here provide an experimental test of handedness of sperm removal by asymmetric genitalia.

## Results and discussion

### Acquired asymmetry

**Figure 1:**
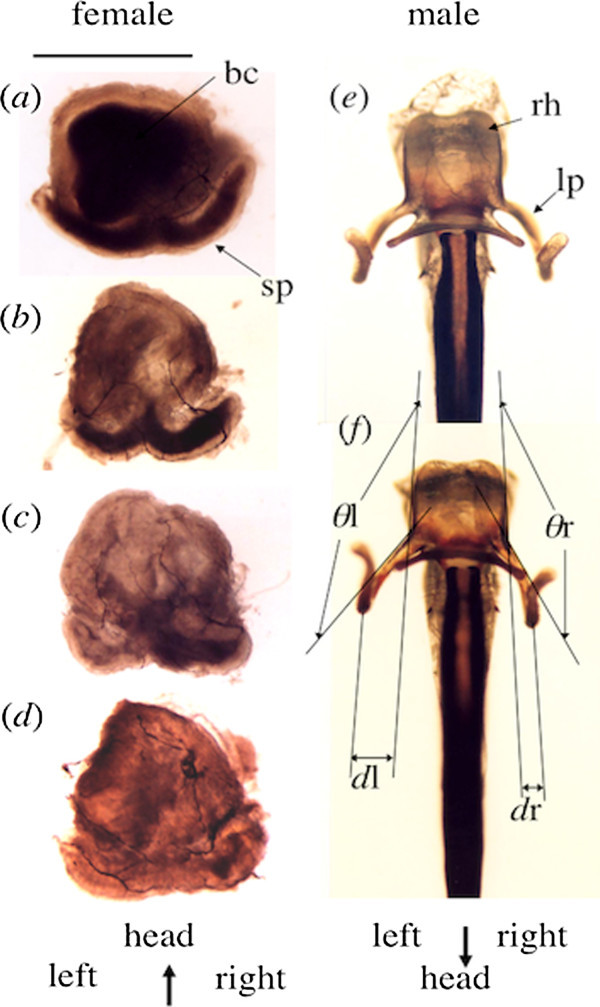
**Female sperm storage organs (** ***a*** **−** ***d*** **, dorsal view) and male genitalia (** ***e*** **−** ***f*** **, ventral view) of** ***Calopteryx cornelia*** **.** The intact sperm mass (darken part) is included in the bursa copulatrix (bc) and entire spermatheca (sp) **(** ***a*** **)**, in the entire spermatheca **(** ***b*** **)**, in the right spermathecal tube **(** ***c*** **)**, and only in the apical part of the right spermathecal tube **(** ***d*** **)**. The male aedeagus consists of the recurved head (rh) and the left and right lateral processes (lp) of immature **(** ***e*** **)** and mature **(** ***f*** **)** males. The angle (*θ*) and outward distance **(** ***d*** **)** of each lateral process were measured. Scale bar is 1 mm.

The right lateral process tended to bend inward with maturity (Figure 1*e*, *f*). The angle ratio of the left and right processes (*θ*l/*θ*r) was 0.990 (*n* = 15, s.d. = 0.041, 95% confidence limit = 0.022) in just emerging males, 0.964 (*n* = 15, s.d. = 0.036, 95% confidence limit = 0.020) in immature males, and 0.928 (*n* = 34, s.d. = 0.031, 95% confidence limit = 0.011) in mature males (ANOVA; *F*_2, 61_ = 17.9, *P* < 0.0001). The degree of outside protrusion of the left process (in the ratio *d*l/*d*r) was 1.342 (*n* = 15, s.d. = 0.402, 95% confidence limit = 0.223) in just emerging males, 1.712 (*n* = 15, s.d. = 0.431, 95% confidence limit = 0.239) in immature males, and 2.468 (*n* = 34, s.d. = 0.759, 95% confidence limit = 0.265) in mature males (ANOVA; *F*_2, 61_ = 19.3, *P* < 0.0001). Thus, the shapes of left and right lateral processes seemed to be symmetric at emergence (nearly *θ*l/*θ*r = 1 and *d*l/*d*r = 1), but became asymmetric with maturation (*θ*l/*θ*r < 1 and *d*l/*d*r > 1).

The morphogenesis of asymmetrical genitalia is unknown. However, the acquisition of strong asymmetries from immature to mature stages may involve two possible mechanisms. One is the structural change accompanied with sclerotisation. Odonata have a relatively long immature (teneral) period after adult eclosion compared to other insect groups and gradually acquire hardness through the maturation process (Corbet 1999). The specimens preserved in 70–99.5% ethanol or dried specimens had an artificially modified shape of genitalia and care must be taken to observe the real shape of genitalia in nature. The other mechanism to bend the lateral process of male genitalia may be a secondary modification by using it at copulation. If the male is behaviourally left-handed, the left process may be used frequently, while the right process is used rarely. This would facilitate stretching out of the left process. However, the observation that no mature males had symmetric genitalia in the field where all males may not experience copulation, suggests that asymmetry is dependent on the acquisition of body hardness.

### Left-handedness in sperm removal

In copulations without interruption, the copulation duration, abdominal movements, and spermathecal sperm number differed among some male manipulation groups, but no consistent effects of the surgical operation on these variables were found (Table 1). In the interrupted copulation, the bursal sperm numbers were much lower than in the copulation without interruption (Table 1), suggesting that bursal sperm removal occurred even if both lateral processes were missing. However, spermathecal sperm were not always emptied (Table 2). When mated with wild males, the left spermathecal tube was emptied more frequently than the right tube; 23 of 40 females had completely emptied left spermatheca but only 3 females had completely emptied right spermatheca (binomial test; *P* < 0.0001). This pattern was replicated in copulation by manipulated control males and by males without only the right lateral process (Table 2). In contrast, when only the left lateral process or both processes were cut, males could not remove any spermathecal sperm (Table 2). These observations indicated that only the left process was functional in spermathecal sperm removal.

**Table 1:** **Effects of male genital treatments on mating behaviour and sperm displacement**

		Normal copulation												Interrupted copulation		
Males	Abbreviation	Copulation duration (s)			Number of abdominal movements			Sperm number in the bursa (×1000)			Sperm number in the spermatheca (×1000)			Sperm number in the bursa (×1000)		
		***n***	***x***	s.d.	***n***	***x***	s.d.	***n***	***x***	s.d.	***n***	***x***	s.d.	***n***	***x***	s.d.
Unmanipulated control (wild)	umc	15	254	147	16	144	60	18	652	404	18	180	138	27	14	13
Manipulated control	mc	8	242	78	8	166	44	8	364	225	8	51	29	8	10	11
Without right process (with left one)	wright	11	144	67	11	90	40	10	672	397	10	75	31	11	72	94
Without left process (with right one)	wleft	7	119	14	8	82	14	8	642	353	8	106	99	12	67	44
Without both processes	wboth	5	143	46	6	91	26	6	852	768	6	43	44	9	66	107
ANOVA		*F*_4, 41_ = 3.9			*F*_4, 44_ = 6.6			*F*_4, 45_ = 1.2			*F*_4, 45_ = 4.3			*F*_4, 62_ = 3.9		
		*P* = 0.009			*P* = 0.0003			*P* = 0.32			*P* = 0.005			*P* = 0.007		
Difference in Tukey’s mct (*P* < 0.05)		umc - wleft			umc - wright			no			umc - mc			no		
					umc - wleft						umc - wboth					
					mc - wright											
					mc - wleft											
					mc - wboth											

ANOVA and Tukey’s multiple comparison tests (statistically different combinations) are also shown.

**Table 2:** **The number of females with nine types of sperm storage organ when copulation was interrupted to allow sperm removal but not to allow ejaculation (remaining sperm masses are shown in black) by control and three types of males with partially cut genitalia**

Males	Sperm-storage organs									Total
										
Unmanipulated control (wild)	2	6	15	1	5	0	1	0	10	40
Manipulated control	1	2	1	0	2	0	1	0	1	8
Without right process (with left one)	0	0	1	4	1	0	0	0	7	13
Without left process (with right one)	0	0	0	0	0	0	0	0	12	12
Without both processes	0	0	0	0	0	0	0	0	9	9

Calopterygid damselflies possess an aedeagus with recurved head to remove bursal sperm and spiny lateral processes to potentially remove spermathecal sperm. However, left and right lateral processes cannot be inserted simultaneously into the Y-shaped spermathecal ducts because they are separated by the recurved head (Figure 1). Observations of female storage organs of pairs fixed during copulation indicated only one process inserted into the spermathecal duct (Córdoba-Aguilar 2002;Cordero Rivera et al. 2004). Such one-handed usage of sperm removal from both sides of Y-shaped spermathecal tubes may concern to the fact that the both spermathecal tubes are not always emptied (e.g., Cordero Rivera et al. 2004;Tsuchiya and Hayashi 2008). Asymmetrical sperm removal ability was first recognised in the central Italian population of *C. haemorrhoidalis* (Cordero Rivera et al. 2004), but not in the Spanish population (Córdoba-Aguilar 1999). The left spermatheca of this Italian population was empty in 19 (61.3%) of 31 females with interrupted copulation but the right spermatheca was empty in only three females (9.7%). Similarly 23 (57.5%) of 40 *C. cornelia* females mated with unmanipulated males had completely emptied left spermatheca and 3 (7.5%) females had the completely emptied right spermatheca. Cordero Rivera et al. (2004) suggested that frequent emptying of the left spermatheca is related to the longer left process than the right, although the mean difference between the left and right lengths is only 0.010 mm (s.e. = 0.004, *n* = 36) of the mean lateral process length of 0.716 mm (range 0.628–0.809). In *C. cornelia*, the left and right lateral processes differ in their shape. In all mature males, the ratio *θ*l/*θ*r was <1 and *d*l/*d*r was >1, suggesting not antisymmetry but directional asymmetry in that the left process was more protruded to the outside. When this protruded lateral process was cut artificially, the males did not remove spermathecal sperm. The folded right process may allow the opposite left process to reach the entrance of the common spermathecal duct and enter into its left duct to remove sperm within it. Thus, the asymmetric shape of the lateral processes may be the most important factor in left-handed sperm removal in *C. cornelia*.

Genital morphology is divergent both intra- and interspecifically in the family Calopterygidae and rapid diversification may be shaped by postmating sexual selection (Cordero Rivera et al. 2004). Females seem to control the ability of males to remove sperm by changing the shape of their sperm storage organs (Córdoba-Aguilar et al. 2003). Movements of male genitalia would be limited in a narrow bursal space, while free in a large bursal space, allowing insertion of the lateral process into the spermathecal duct. In the former case, asymmetric male genitalia would be expected, as found in this study; one side of the lateral processes is folded to bear some space in the bursa. To determine how common asymmetric male genitalia are in calopterygid damselflies, reexamining their genitalia in relation to female sperm storage strategies will be necessary.

## Conclusions

We found left-handed sperm removal by male *Calopteryx cornelia* (Odonata: Calopterygidae). Males remove rival sperm stored in the female spherical bursa copulatrix using a recurved head of the aedeagus, and those stored in the Y-shaped tubular spermatheca using a pair of spiny lateral processes of the aedeagus prior to their own ejaculation to prevent sperm competition. However, the left and right lateral processes were asymmetric; the left lateral process was more developed than the right process, and only the left process functioned in spermathecal sperm removal. In fact, sperm stored in the right spermathecal tube were rarely removed by the male. To determine how common such asymmetric male genitalia are in calopterygid damselflies, we need reexamination of male genital shape in the previous studies.

## Methods

### Morphological measurements

A total of 64 males were collected at the Yozawa and Hirai Rivers, the neighbouring small tributaries of the Tama River in Tokyo, central Japan (35°45′N, 139°11′E), from May to September in 2008 and 2009. Maturity was identified by wing hardness and colour of the ventral side of abdominal tips; just emerging males with soft wings and a yellowish abdominal spot, immature males with harder wings and a yellowish abdominal spot, and mature males with hard wings and a whitish abdominal spot. After collection, males were anaesthetised with CO_2_ and their genitalia were dissected out. Photographs from the ventral side of removed genitalia were taken under a binocular microscope (MZ FLIII, Leica, Wetzlar, Germany). The angle and outward distance of each lateral process from the edge of the recurved head (*θ* and *d* in Figure 1, respectively) were measured on the photographs to calculate the degree of genital asymmetry as *θ*l/*θ*r and *d*l/*d*r, respectively.

### Hand-pairing and sperm counting

Mature individuals were used for the mating experiments in the mid-reproductive season (mid-July to early August) in 2003–2008. All females used for experiments included sperm in their storage organs, i.e., they were non-virgin females. We used hand-pairing techniques to mate damselflies in the field (Opphenheimer and Waage 1987). The thorax of a female was tethered with fine gauge nylon monofilament (0.104 mm in diameter, 0.50 m in length). The opposite end of the filament was tied to the tip of a rod (*ca*. 10 mm in diameter, 0.7 m in length). When the male attempted to grasp the female and tandem linkage was achieved, the wings of the male and female were gently released. After flying to nearby vegetation within the length of the nylon filament, the male translocated sperm to its sperm vesicles and copulated. Copulation consists of three distinct behavioural stages in Calopterygidae (Miller and Miller 1981). The first stage involves slow, rhythmic movements of the male abdomen (sperm removal stage). The second stage is quiescent and relatively short and constant regardless of the duration of copulation (transition), while the third stage consists of a series of rapid, shallow rhythmic movements of the male abdomen (sperm transfer stage). In examinations of sperm displacement, we interrupted a copulating pair soon after initiation of the second stage to prevent sperm transfer by the mating male after sperm displacement. The mean total number of abdominal movements to interruption was 92.9 (*n* = 82, s.d. = 14.2).

Individuals were kept in an envelope in a cool, dark container and transported to the laboratory. Females were anaesthetised with CO_2_ and dissected within 12 h of collection. Intact sperm storage organs were then removed and placed in Grace’s insect cell culture medium (Gibco-BRL, Life Technologies, New York, NY, USA) on glass plates. The apparent degree of sperm removal in each spermathecal duct was distinguished into three categories; no removal, partial removal, and complete removal (Figure 1*a*-*d*). After recording these categories of sperm displacement of each spermathecal duct, the bursa copulatrix was removed from the spermatheca using fine forceps and placed in a tube containing 50 μl of fresh Grace’s buffer. Sperm in the bursa were mixed in the buffer by gentle pipetting. Sperm suspensions were diluted 1×, 10×, or 50× according to their apparent concentrations. The total number of sperm in a given tube was estimated by counting all sperm in 1 μl. After vortexing, aliquots of 1 μl of the sperm suspension were placed onto glass slides and dried at room temperature for several days. Samples on slides were then fixed in 99.5% ethanol for 10 min, dried again for approximately 20 min, placed in 5% Giemsa solution for 30 min, washed in distilled water for 5 min, covered with a cover glass and observed under a light microscope (100×). We averaged the total number of sperm from five separate 1-μl drops per organ.

### Surgical removal of lateral processes

The genitalia of experimental males were lifted out of the genital cavity, each or both of the lateral processes were severed at the basal position and the operated genitalia were retracted to the genital chamber using fine forceps under a binocular stereoscope (10–15×; 216031, Olympus, Tokyo, Japan). As this procedure only took approximately 30 s, males were not anaesthetised. The lateral processes are sclerotised and can be cut without effusion of haemolymph. Males were marked with individual numbers on their wings using paint markers and then released near their capture site. In the manipulated control males, the genitalia were treated as in experimental males, but each lateral process was tapped ten times with fine forceps under the binocular stereoscope. Females were mated by hand with one of an experimental, manipulated control, and unmanipulated control (wild) male. Paired males and females were all captured and transported to the laboratory for dissection. Any male was never used more than once.

To examine the effects of male surgical treatments on copulation, we compared normal pairs that were hand-mated but not interrupted until the end of copulation. In this case, we recorded the total number of male abdominal movements, the duration of copulation from genital contact to release and sperm in the bursa and spermatheca. The number of pairs examined were 18 for wild controls, 8 for manipulated controls, 11 for pairing of males without the right process, 8 for pairing of males without the left process, and 6 for pairing of males without both processes, although a few values were missing due to difficulties in measuring them in the field or failure of sperm count in the laboratory.
